# A Collision Cross Section Database for Extractables and Leachables from Food Contact Materials

**DOI:** 10.1021/acs.jafc.2c00724

**Published:** 2022-04-05

**Authors:** Xue-Chao Song, Elena Canellas, Nicola Dreolin, Jeff Goshawk, Cristina Nerin

**Affiliations:** † Department of Analytical Chemistry, Aragon Institute of Engineering Research I3A, EINA, University of Zaragoza, Maria de Luna 3, 50018 Zaragoza, Spain; ‡ Waters Corporation, Altrincham Road, SK9 4AX Wilmslow, United Kingdom

**Keywords:** ion mobility, collision cross section, food safety, food contact materials, extractables, leachables

## Abstract

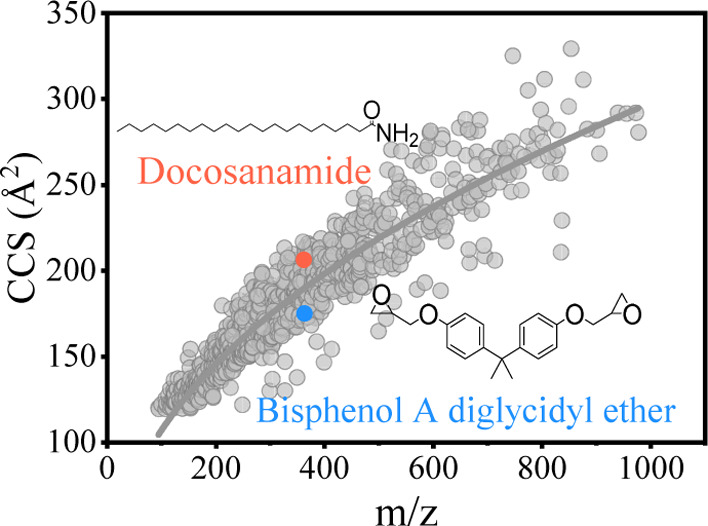

The chemicals in food contact materials (FCMs) can migrate into food and endanger human health. In this study, we developed a database of traveling wave collision cross section in nitrogen (^TW^CCS_N2_) values for extractables and leachables from FCMs. The database contains a total of 1038 ^TW^CCS_N2_ values from 675 standards including those commonly used additives and nonintentionally added substances in FCMs. The ^TW^CCS_N2_ values in the database were compared to previously published values, and 85.7, 87.7, and 64.9% [M + H]^+^, [M + Na]^+^, and [M – H]^−^ adducts showed deviations <2%, with the presence of protomers, post-ion mobility spectrometry dissociation of noncovalent clusters and inconsistent calibration are possible sources of CCS deviations. Our experimental ^TW^CCS_N2_ values were also compared to CCS values from three prediction tools. Of the three, CCSondemand gave the most accurate predictions. The ^TW^CCS_N2_ database developed will aid the identification and differentiation of chemicals from FCMs in targeted and untargeted analysis.

## Introduction

Food contact materials (FCMs) are important sources of contaminations of the food. The chemical constituents of FCMs, termed food contact chemicals (FCCs), can be classified into two categories: intentionally added substances (IAS) and non-IAS (NIAS). IAS include known additives, including plasticizers, antioxidants, photoinitiators, lubricants, and slip agents, that are added to FCMs during processing in order to confer favorable characteristics and extend service life. NIAS can be broadly grouped into three categories: side products, breakdown products, and contaminants. Side products form due to the incomplete polymerization of starting substances^1^ or the interaction between migrants and food.^2^ Breakdown products arise from the degradation of polymers and additives during manufacture and use.^3,4^ The origins of contaminants include the manufacturing process, shelf life, and the recycling process.^5,6^ All these compounds can potentially migrate into food and pose a risk to the health of consumers.^7−9^

It is challenging to achieve a full identification of FCCs in FCMs due to the high complexity of matrices. In the study by Zimmermann et al.,^10^ only ∼8% of detected features were identified by ultrahigh performance liquid chromatography coupled to a quadrupole-time-of-flight mass spectrometer (UPLC-QToF), indicating that most of the chemicals from plastics remain unknown. Regulation (EC) no. 1935/2004 establishes that the substances in FCMs cannot migrate into food in quantities large enough to endanger human health.^11^ Therefore, the FCCs in FCMs must be identified and quantified.

The coupling of ion mobility spectrometry (IMS) with HRMS provides a powerful tool for the identification and separation of small molecules commonly found in the food industry and environmental analyses, including mycotoxins,^12,13^ pesticides,^14,15^ drug and drug-like compounds,^16^ phenolics,^17,18^ and FCCs.^19,20^ It has been reported that some structural isomers^21^ and stereoisomers^22^ can be separated by IMS. Collision cross section (CCS) is a parameter derived from the drift time (DT) using a power-law calibration for the traveling wave IMS (TWIMS) device. CCS measurement is consistent across different IMS platforms and laboratories.^13,23,24^ Hinnenkamp et al.^23^ compared the CCS values determined by TWIMS and drift tube IMS (DTIMS), finding that 93% of protonated adducts and 87% of sodiated adducts have deviations in the CCS values lower than 2%. The study of Righetti et al.^13^ indicated that the ^TW^CCS_N2_ measurements of all ion species showed the deviations of less than 1.5% between two Vion platforms from different laboratories. Additionally, the deviation of ^TW^CCS_N2_ values was within 2% for 96.4% of ions measured on Vion and Synapt platforms. The high reproducibility of CCS makes it a reliable parameter for inclusion in mass-spectral libraries. In addition to RT and fragment ion information, including CCS data in the identification process will improve confidence, thereby reducing the number of tentative identifications.

Recently, several open-source, experimental CCS databases have been constructed for mycotoxins,^12^ steroids,^25^ phenolics,^26^ pesticides,^14,27^ drugs and drug-like compounds,^16^ and organic environmental micropollutants.^28−30^ Additionally, some research groups have developed machine-learning-based tools to predict the CCS values of molecules. These include CCSondemand,^31^ AllCCS,^32^ CCSbase,^33^ and DeepCCS.^34^

In the previous study,^35^ 635 traveling wave CCS in nitrogen (^TW^CCS_N2_) values from 488 standards were measured in the positive ion mode, and much effect was only focused on the development of a CCS prediction tool, the ^TW^CCS_N2_ values in the negative mode were not measured and the ^TW^CCS_N2_ distributions of different types of additives were not investigated. Thus, the goal of this study was to build a more comprehensive ^TW^CCS_N2_ database for extractables and leachables found in FCMs in both positive and negative ion modes, comprising commonly used additives (e.g., plasticizers, antioxidants, photoinitiators, and lubricants) and NIAS (degradation products of additives and oligomers). The ^TW^CCS_N2_ values in our database were compared to previously published CCS measurements and reasons explaining high deviations, in some instances, are discussed. In addition, the experimental ^TW^CCS_N2_ values in our database were compared to the predicted CCS values from three prediction tools in order to evaluate the applicability of CCS prediction tools in the field of FCMs.

## Materials and Methods

### Chemicals and Reagents

Standards of commonly used additives in FCMs, including plasticizers, antioxidants, photoinitiator, UV absorbers, slip agent, lubricants, and degradation products of additives were purchased from Sigma-Aldrich Quimica S.A. (Madrid, Spain), Extrasynthese (Genay, France), and Cayman Chemical Company (Ann Arbor, Michigan, USA). Oligomers of adhesives, polyamide (PA), and polylactic acid (PLA) were isolated from associated polymers in our laboratory. The standard stock solutions at a concentration of 1000 mg kg^–1^ were prepared by dissolving 10 mg of standards with 10 g of methanol using an electronic accurate balance from Mettler Toledo (XS205, 0.1 mg, Greifensee, Switzerland). If the standards were not dissolved in methanol, other solvents, such as ethanol, dichloromethane, or dimethyl sulfoxide, were used. The measured ^TW^CCS_N2_ values would not be affected by the solvents, as it is independent from sample matrices.^12^ The working solutions at ∼1 mg kg^–1^ were prepared by the dilution of 10 μL of stock solution with 10 mL of methanol. Each working solution contained a mixture of 8–10 analytes and all the mixtures were kept in the dark at −20 °C until analysis.

HPLC grade methanol (≥99.9%), ethanol (≥99.9%), dichloromethane (≥99.8%), and dimethyl sulfoxide (≥99.8%) were purchased from Scharlau Chemie S.A (Sentmenat, Spain). Ultrapure water was produced by a Millipore Milli-QPLUS 185 system (Madrid, Spain).

### UPLC-IMS-QToF Analysis

The working solutions at ∼1 mg kg^–1^ were measured using an Acquity I-Class UPLC system coupled to a Vion IMS-QToF mass spectrometer (Waters, Manchester, UK). Detailed setup conditions and parameters for the Vion, CCS calibration functions are given in the Supporting Information.

The Major Mix IMS/ToF calibration kit (ref. 186008113) from Waters (Manchester, UK) was used for the CCS calibration. The calibration compounds and their CCS values in positive and negative ionization modes are shown in Tables S1 and S2, respectively. In the positive ion mode, the TWIMS platform was calibrated with polyalanine and nine drug-like compounds with a *m*/*z* range of 151.1–1154.6 Da and a CCS range of 130.4–333.6 Å^2^. In the negative ion mode, two fluoroalkanoic acids were added in the calibration mix, providing a *m*/*z* range of 151.1–1167.0 Da and a CCS range of 130.1–322.4 Å^2^. A quality control (QC) solution (Vion Test Mix, ref. 186008462) from Waters (Manchester, UK) was systematically injected before and after each batch of standard solutions to monitor the system stability. Detailed information about the nine compounds in QC solution is shown in Table S3. The variations of the *m*/*z* and ^TW^CCS_N2_ measurements for the QC solution were less than 5 ppm and 2%, respectively.

Data acquisition and processing were performed on UNIFI v.1.9.4. (Waters Corp.). Only the ^TW^CCS_N2_ values of singly charged ions were considered and included values for [M + H]^+^, [M + Na]^+^, [M + NH_4_]^+^, [M – Na + 2H]^+^, and [M – Cl]^+^ in the positive mode and [M – H]^−^ and [M + HCOO]^−^ in the negative mode.

### Precision of ^TW^CCS_N2_ Measurement

In order to validate the interday precision of ^TW^CCS_N2_ measurements, a mixed standard solution at ∼1 mg kg^–1^ containing 38 representative IAS and NIAS in FCMs was injected once a week over a period of 2 months. The mixed standard solution contained plasticizers, antioxidants, photoinitiators, bisphenols, and common degradation products, such as 3,5-di-*tert*-butyl-4-hydroxybenzaldehyde. Detailed information on these compounds is provided in Table S4.

### Comparison with Published CCS Measurements

The comparison between ^TW^CCS_N2_ values in our database and those obtained from the literature is crucial to determine whether our database could be used across different laboratories and instrumental types. Hence, several CCS databases and publications were consulted for reference CCS values of some of the compounds considered in this study.^13,14,16,23,25,27−30,36−41^ The CCS deviations (ΔCCS%) were calculated using the ^TW^CCS_N2_ values in our database as the reference values.

### Evaluation of Public CCS Prediction Tools

CCS values predicted by machine learning algorithms can be used when empirical CCS values are not available. To evaluate the accuracy of existing CCS prediction tools for FCCs, the ^TW^CCS_N2_ values of the compounds in our database were compared against those generated by three CCS prediction tools: AllCCS (http://allccs.zhulab.cn/) proposed by Zhou et al.,^32^ CCSbase (https://ccsbase.net/) from Libin Xu Lab,^33^ and CCSondemand (https://ccs.on-demand.waters.com/) from Broeckling and co-workers.^31^

## Results and Discussion

### CCS Deviations of QC Compounds

The ^TW^CCS_N2_ database was built over the period from November 2018 to July 2021. A total of 76 and 24 batches of QC solutions were injected in the positive and negative modes, respectively, during the database creation. The comparison between reference and experimental CCS values for QC compounds is shown in Table S5, and the distributions of their CCS deviations are shown in Figure S1. It can be seen that for both ion modes, the average CCS variation was less than 1.1% and the relative standard deviations (RSDs) ranged from 0.5% to 0.8%. These data indicate a high degree of accuracy and reproducibility of the ^TW^CCS_N2_ measurements over the course of almost 3 years. Acetaminophen presented relatively high CCS deviations in both ion modes, which is possibly due to its low *m*/*z* and CCS values. This observation highlights the importance of adding more data points to the calibration curve for *m*/*z* values below 150.

### ^TW^CCS_N2_ Database Overview

**Figure 1:**
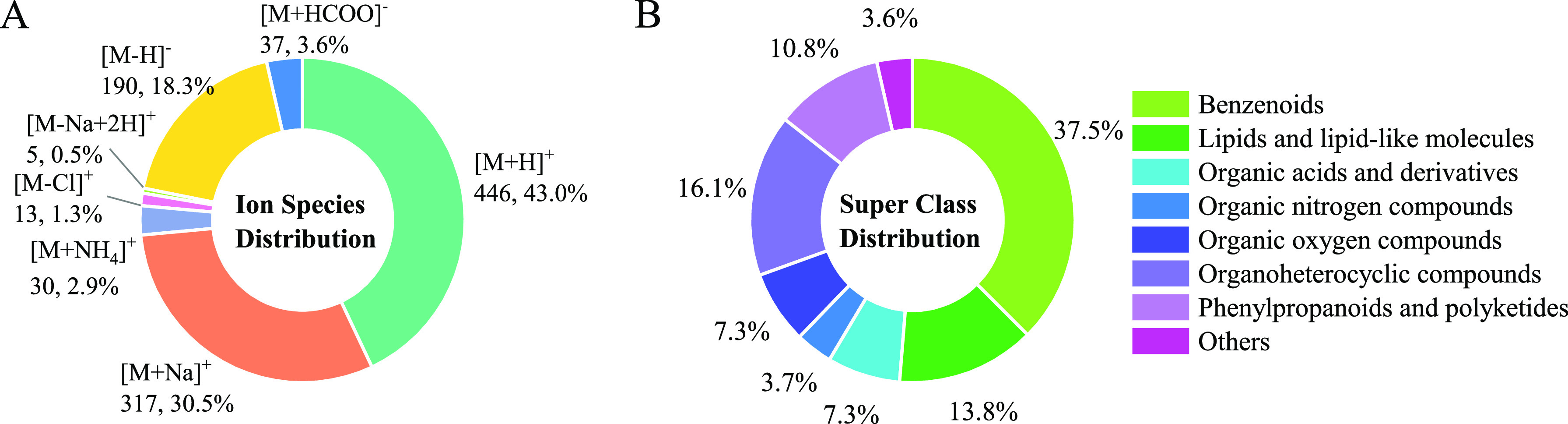
(A) Distribution of the 1038 measured ions from positive and negative ionization modes; (B) distribution of 675 detected compounds across super classes.

This work presents a ^TW^CCS_N2_ database with respect to extractables and leachables in FCMs, which consists of commonly used additives, degradation products of additives, oligomers, and natural phenolic compounds from antioxidant active packaging. Detailed information about the chemicals in the database, such as compound name, adduct, monoisotopic mass, molecular formula, canonical SMILES, InChIKey, and class can be seen in the Supporting Information. Figure 1A shows that a total of 1038 ions were detected for the 675 standards analyzed. The detected ions could be divided into two groups of 811 cations (446 [M + H]^+^, 317 [M + Na]^+^, 30 [M + NH_4_]^+^, 5 [M – Na + 2H]^+^, and 13 [M – Cl]^+^) and 227 anions (190 [M – H]^−^ and 37 [M + HCOO]^−^). In the positive ion mode, 580 compounds were detected, including the commonly used plasticizers, antioxidants, photoinitiators, primary aromatic amines, slip agents, and oligomers. These compounds contain either carbonyl oxygen, amine, or ether oxygen in their structure. In the negative ion mode, 205 compounds were detected, which included lubricants, hindered phenol antioxidants, bisphenols, and perfluoroalkyl substances (PFAS).

The super classes of the 675 standards analyzed were obtained using ClassyFire,^42^ and the distribution of classes is shown in Figure 1B. More compounds belong to the benzenoid super class than any other class in the database. This is unsurprising because commonly used additives in FCMs, such as antioxidants, biocides, bisphenols, nucleating agents, photoinitiators, phthalate-based plasticizers, and UV absorbers belong to this super class. Lipid and lipid-like molecules and organoheterocyclic compounds also account for a large part of the database. The former contains lubricants, adipate-based and sebacate-based plasticizers, slip agents, and fatty acid esters. The latter includes colorants, pesticides, drug-like compounds, and UV absorbers.

**Figure 2:**
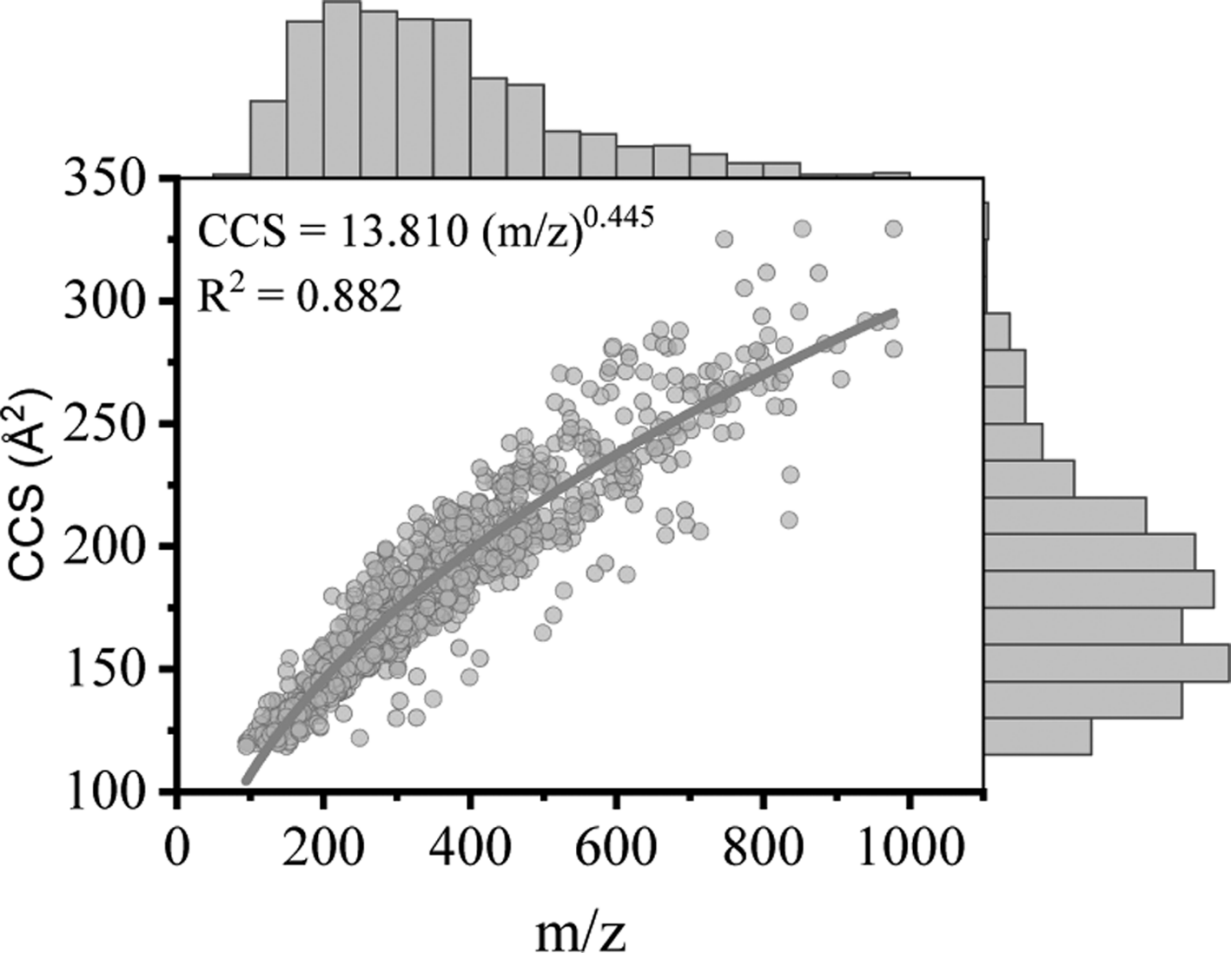
Depiction of ^TW^CCS_N2_ values vs *m*/*z* values for 1038 ions together with the distribution of ^TW^CCS_N2_ and *m*/*z* values.

A depiction of ^TW^CCS_N2_ versus *m*/*z* for 1038 ions and the distribution of ^TW^CCS_N2_ and *m*/*z* values are shown together in Figure 2. The correlation between ^TW^CCS_N2_ and *m*/*z* was described by the power regression model, with *R*^2^ of 0.882. ^TW^CCS_N2_ values range from 119.6 Å^2^ ([M + H]^+^ of benzaldehyde) to 329.4 Å^2^ ([M + H]^+^ of 3,9-Bis(2,4-dicumylphenoxy)-2,4,8,10-tetraoxa-3,9-diphosphaspiro[5.5]undecane) and *m*/*z* values range from 94 Da ([M + H]^+^ of aniline) to 977 Da ([M + Na]^+^ of PLA 13). Figure 2 shows that 95% of the measured ^TW^CCS_N2_ values are accounted for in the *m*/*z* region from 93 to 700 Da. ^TW^CCS_N2_ values are mainly distributed in the range of 119–220 Å^2^ which accounts for 83.3% of the measured ^TW^CCS_N2_ values. Besides, 93.3% (968 out of 1038) of ^TW^CCS_N2_ values located in the calibration range, the other 70 ^TW^CCS_N2_ values were below the lowest CCS values in calibrates (130.4 Å^2^ in the positive mode and 130.1 Å^2^ in the negative mode).

### CCS Distribution of Commonly Observed Additives and NIAS

**Figure 3:**
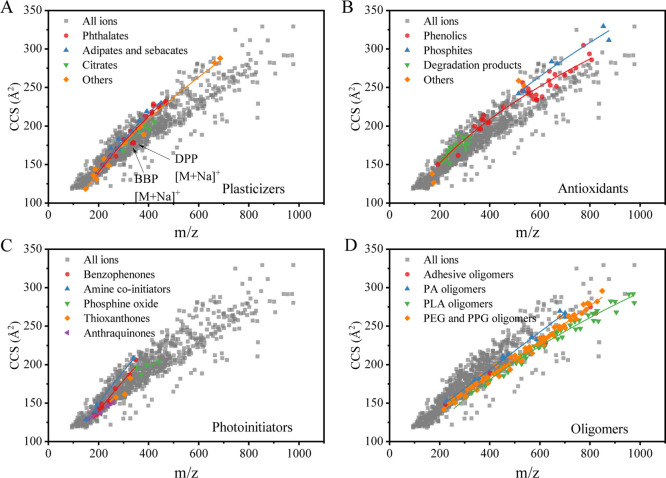
Depiction of ^TW^CCS_N2_ values vs *m*/*z* values for common additives and NIAS in FCMs: (A) plasticizers; (B) antioxidants; (C) photoinitiators; and (D) oligomers. BBP: benzyl butyl phthalate, DPP: diphenyl phthalate.

Because CCS is a measurement related to the size, shape, and charge of an ion,^43^ different relationships between CCS versus *m*/*z* have been observed for compounds presenting different structural characteristics.^16,25,28^ Correlations between the CCS and *m*/*z* of the commonly used additives (plasticizers, antioxidants, and photoinitiators) and oligomers studied here are shown in Figure 3, and their regression equations are shown in Table S6. Figure 3A presents the ^TW^CCS_N2_ versus *m*/*z* relationship of 57 ions from 49 plasticizers, with different colors denoting different types of plasticizers. It is evident that the trend line for adipates and sebacate-based plasticizers (10 [M + Na]^+^ ions) have a steeper gradient than the trend lines for phthalates (6 [M + H]^+^ and 24 [M + Na]^+^ ions) and citrates (2 [M + H]^+^ and 3 [M + Na]^+^ ions). Adipates and sebacate-based plasticizers appear to have a more elongated structure due to their linear-chain molecules, which leads to a larger rotationally averaged collision area for a given *m*/*z*. The trend line for phthalates (i.e., diesters of *ortho*-phthalic acid) has a shallower gradient. In general, the structures of this class of plasticizers contain both aryl and alkyl groups (e.g., dibutyl phthalate) and the compact aryl group will lead to a smaller ^TW^CCS_N2_ value. This is supported by the lower ^TW^CCS_N2_ values of benzyl butyl phthalate (BBP) and diphenyl phthalate (DPP) when compared to the ^TW^CCS_N2_ values of other phthalates with a similar *m*/*z*. In the structures of these two compounds, one or two alkyl groups are replaced by aryl groups. Citrates have relatively lower ^TW^CCS_N2_ values compared to phthalates, adipates, and sebacate-based plasticizers with similar *m*/*z* values. This is may be due to the compact side chains in their structures, as demonstrated by Belva et al.^28^

The presence of branched alkyl groups in phthalates produces various structural isomers. To study the effect of alkyl groups on the conformation of phthalates, the ^TW^CCS_N2_ values of eight phthalates, with either linear or branched alkyl groups, were measured in triplicate and the average ^TW^CCS_N2_ values together with their standard deviations are presented in Table 1. It can be seen that diisoalkyl phthalates have slightly lower ^TW^CCS_N2_ values compared to the ^TW^CCS_N2_ values for corresponding dialkyl phthalates. Compare, for example, dipropyl phthalate (171.79 ± 0.15 Å^2^) and diisopropyl phthalate (170.65 ± 0.07 Å^2^). This indicates that the branched alkyl group can lead to a slightly more compact molecule. The CCS deviations between diisoalkyl phthalates and dialkyl phthalates, though, were less than 1.5 Å^2^ which is lower than variations in the ^TW^CCS_N2_ values observed for other molecules obtained using TWIMS platforms (±2%).^13,25,44^ As such, the definitive identification of such isomers may require a IMS device with higher resolving power and better reproducibility (providing CCS deviations < 0.5%).

**Table 1:** ^TW^CCS_N2_ Values of Sodiated Adducts of Isomeric Phthalate-Based Plasticizers (*n* = 3)

compounds	*m*/*z*	RT (min)	^TW^CCS_N2_ ± SD (Å^2^)	RSD (%)
dipropyl phthalate	273.1097	6.23	171.79 ± 0.15	0.09
diisopropyl phthalate	273.1097	6.13	170.65 ± 0.07	0.04
dibutyl phthalate	301.1410	6.88	183.61 ± 0.03	0.02
diisobutyl phthalate	301.1410	6.80	182.13 ± 0.12	0.06
dinonyl phthalate	441.2975	8.42	226.35 ± 0.13	0.06
diisononyl phthalate	441.2975	8.37	225.13 ± 0.09	0.04
didecyl phthalate	469.3288	9.01	233.92 ± 0.24	0.10
diisodecyl phthalate	469.3288	8.65	232.47 ± 0.03	0.01

Diesters of isophthalic acid and terephthalic acid can also lead to the presence of isomers. The ^TW^CCS_N2_ values of three phthalate-based plasticizers, bis(2-ethylhexyl) phthalate, bis(2-ethylhexyl) isophthalate, and bis(2-ethylhexyl) terephthalate were measured as 218.42 ± 0.31 Å^2^, 218.45 ± 0.14 Å^2^, and 218.05 ± 0.20 Å^2^, respectively. Their ^TW^CCS_N2_ values were not significantly different, possibly because their molecules are flexible, and interact with the drift gas in a similar manner. This type of isomers cannot be separated using TWIMS systems with Rp below 60 full width at half-maximum (FWHM).^45^

^TW^CCS_N2_ values of other types of plasticizers (6 [M + H]^+^ and 6 [M + Na]^+^ ions), such as benzoates and organophosphates, were also included in the database. Tris(2,4-di-*tert*-butylphenyl) phosphate has the highest ^TW^CCS_N2_ value of all the plasticizers, with values of 282.1 Å^2^ for [M + H]^+^ and 287.9 Å^2^ for [M + Na]^+^. It should be mentioned that this compound is also an oxidation product of Irgafos 168 and can be used as a flame retardant.

A total of 67 ^TW^CCS_N2_ values were obtained from 38 antioxidants and their degradation products. Two categories of antioxidants were included in the data set, hindered phenols (5 [M + H]^+^, 14 [M + Na]^+^, 12 [M – H]^−^, 2 [M + NH_4_]^+^, and 1 [M + HCOO]^−^ ions) and phosphites (3 [M + H]^+^ and 3 [M + Na]^+^ ions). The former category is primary antioxidants, which can eliminate free radicals, and the latter category can decompose hydroperoxide, working as secondary antioxidants.^46^ The relationship between ^TW^CCS_N2_ and *m*/*z* values of phenol antioxidants can be described by a power model with a determination coefficient *R*^2^ = 0.936. The ^TW^CCS_N2_ values of the phenols ranged from 150.3 to 305.2 Å^2^ and those for the phosphites ranged from 241.9 to 329.4 Å^2^.

Many degradation products can be generated by the oxidation of antioxidants and are an important set of NIAS in FCMs. A total of 23 ^TW^CCS_N2_ values were obtained for degradation products, including 11 [M + H]^+^, 7 [M + Na]^+^, 4 [M – H]^−^, and 1 [M + HCOO]^−^. The relationship between their ^TW^CCS_N2_ and *m*/*z* values is depicted in Figure 3B. 2,6-Di-*tert*-butyl-1,4-benzoquinone ([M + H]^+^ 156.0 Å^2^, [M + Na]^+^ 171.2 Å^2^) and 3,5-di-*tert*-butyl-4-hydroxybenzaldehyde ([M – H]^−^ 162.7 Å^2^, [M + H]^+^ 164.9 Å^2^) are degradation products of butylated hydroxytoluene (BHT).^47^ 3-(3,5-Di-*tert*-butyl-4-hydroxyphenyl)propionic acid ([M + Na]^+^ 175.6 Å^2^) can be produced from Irganox 245, Irganox 1076, Irganox 1035, and Irganox 1098. This compound can be further oxidized into 7,9-di-*tert*-butyl-1-oxaspiro(4,5)deca-6,9-diene-2,8-dione ([M + H]^+^ 173.9 Å^2^, [M + Na]^+^ 185.2 Å^2^).^48^ Many of the ^TW^CCS_N2_ values of degradation products are reported here for the first time, which contribute a lot to the application of IMS in the analysis of FCCs.

There are 33 ^TW^CCS_N2_ values for 24 photoinitiators included in the ^TW^CCS_N2_ database. Their ^TW^CCS_N2_ values range from 129.2 {[M + H]^+^ of 4-(dimethylamino)benzaldehyde} to 208.2 Å^2^ ([M + H]^+^ of 4-octadecylmorpholine). The photoinitiators are classified into benzophenones, amine co-initiators, phosphine oxides, thioxanthones, and anthraquinones based on their structural characteristics. The relationship between the ^TW^CCS_N2_ and *m*/*z* values for the photoinitiators is shown in Figure 3C. The trend line for amine co-initiators (4 [M + H]^+^ ions) has a slightly higher gradient than the other classes due to the high ^TW^CCS_N2_ values of 4-octadecylmorpholine. This compound contains an octadecyl group which is likely to increase the number of collisions of the molecule with the drift gas. Thioxanthones (3 [M + H]^+^ and 1 [M + Na]^+^ ions) and anthraquinones (6 [M + H]^+^ ions) have slightly lower ^TW^CCS_N2_ values than benzophenones, possibly due to the presence of additional rings in their molecular structures, as shown in Figure S2. The trend line for phosphine oxide (1 [M + H]^+^, 2 [M + H]^+^ and 1 [M + HCOO]^−^) has a relatively shallow gradient which is most likely due to the multiple phenyl groups in the structures of these molecules.

Oligomers are an important source of NIAS and 130 ^TW^CCS_N2_ values from 56 oligomers of five types are included in the ^TW^CCS_N2_ database. The relationship between ^TW^CCS_N2_ and *m*/*z* values for the oligomers is shown in Figure 3D. Adhesive oligomers are products of reactions between adipic acid and 1,4-butanediol. PA oligomers originate from two different polymers: PA6 (a polymer of caprolactam) and PA66 (a polymer of 1,6-diaminohexane and adipic acid). The structure of PLA oligomers can be either linear or cyclic. The structures of polyethylene glycol (PEG) and polypropylene glycol (PPG) oligomers are similar so they are represented by the same color in Figure 3D. For the PA (8 [M + H]^+^ and 8 [M + Na]^+^ ions) and the PEG and PPG oligomers (24 [M + H]^+^, 23 [M + Na]^+^ and 18 [M + NH_4_]^+^ ions), the relationship between ^TW^CCS_N2_ and *m*/*z* values followed linear regression models, with *R*^2^ values of 0.993 and 0.984, respectively. The ^TW^CCS_N2_ and *m*/*z* relationship for PLA oligomers (13 [M + H]^+^, 18 [M + Na]^+^, and 11 [M + NH_4_]^+^ ions) followed a power model, with a *R*^2^ value of 0.975. Adhesive, PA, and PLA oligomers belong to the super class of phenylpropanoids and polyketides, while PEG and PPG belong to the organic oxygen compounds class. 35 ions for the oligomers had ^TW^CCS_N2_ values above 250 Å^2^ and *m*/*z* values above 700 Da, which expanded the chemical space covered by the ^TW^CCS_N2_ database.

**Figure 4:**
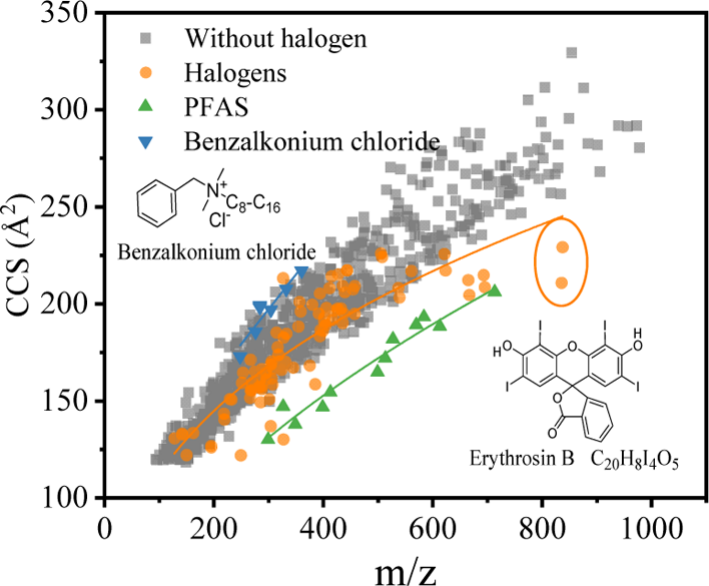
Depiction of ^TW^CCS_N2_ values vs *m*/*z* values for halogenated compounds.

Flame retardants, lubricants, and slip agents are also commonly used additives in plastics and the ^TW^CCS_N2_ values for those measured for this study are shown in Figure S3. Lubricants mainly contain long-chain fatty acids and slip agents mainly contain long-chain fatty amides. As such, it is understandable that these two types of additives have relatively high ^TW^CCS_N2_ values. Plotting the ^TW^CCS_N2_ values against the *m*/*z* values does not reveal any specific patterns for flame retardants. Dibutyl phosphate and tributyl phosphate have high ^TW^CCS_N2_ values with respect to *m*/*z* due to the presence of alkyl groups, tris(2-chloroethyl) phosphate and chlorendic acid have low ^TW^CCS_N2_ values with respect to *m*/*z* due to the presence of chlorine, while tri-p-cresyl phosphate and octicizer reside between the two other groups. On plotting the ^TW^CCS_N2_ values against the *m*/*z* values for halogenated compounds, the resulting trend line tends to be different from that for compounds only containing C, H, O, N, S, and P.^28^ To clearly show the effect of halogens on CCS values, the ^TW^CCS_N2_ distributions of halogenated compounds are shown in Figure 4. A total of 114 ions from 81 halogenated compounds were included in the ^TW^CCS_N2_ database, and it is clear that their CCS values tend to be lower for a given *m*/*z* across the *m*/*z* range. PFAS and benzalkonium chloride are two types of surfactants used in plastic products. It should be mentioned that benzalkonium chlorides appear in the positive ion mode as benzalkonium cations, although they cannot be strictly classified in the halogenated compounds. However, the comparison of their CCS distribution with that of PFAS can clearly show the effect of halogens on CCS values. PFAS compounds contain carbon-fluorine bonds, and their ^TW^CCS_N2_ values are much lower in general than other compounds of similar *m*/*z* values. By contrast, ^TW^CCS_N2_ values of benzalkonium chloride tend to be high for a given *m*/*z* because these compounds contain alkyl groups and will lose chloride in positive ion mode mass spectrometry.

The interday precision of ^TW^CCS_N2_ measurements was monitored using 38 FCCs over the course of 2 months within which the IMS cell was calibrated twice. 70 ions were detected including 15 [M + H]^+^, 23 [M + Na]^+^, 28 [M – H]^−^, and 4 [M + HCOO]^−^, and the distribution of the RSDs of the ^TW^CCS_N2_ measurements of these ions is shown in Figure S4. Excellent interday precision was obtained with all RSD values lower than 0.7%. 85.7% (60/70) of adducts had RSD values in the range of 0.3–0.5%. Similar interday precision of TWIMS platform has also been shown by Regueiro et al.,^27^ with most RSDs in their work ranging from 0.3 to 0.5%.

### Comparison with Existing Literature CCS Values

**Figure 5:**
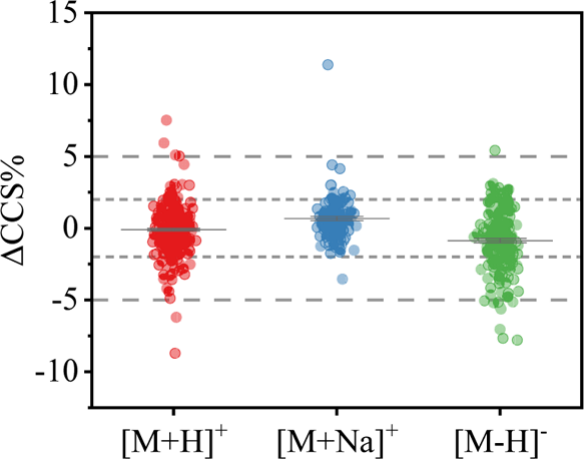
Comparing ^TW^CCS_N2_ values in the database with published CCS values.

In order to check the accuracy of our ^TW^CCS_N2_ data and ensure that it is independent of the IMS platform and laboratory used to acquire it, the ^TW^CCS_N2_ values of the three main adducts ([M + H]^+^, [M + Na]^+^, [M – H]^−^) were compared to previously published CCS values. CCS deviations (ΔCCS%) were calculated using the ^TW^CCS_N2_ values in our database as the reference, and the results are shown in Figure 5, The CCS records with deviations higher than 5% are shown in Table S7.

For some compounds, several CCS records can be found in different publications. A total of 300, 144, and 208 CCS measurements were found for 123 [M + H]^+^, 71 [M + Na]^+^, and 93 [M – H]^−^ adducts, respectively. It is usual to use a tolerance of ±2.0% for CCS measurements in IMS analysis.^13^ 85.7, 87.7, and 64.9% of the [M + H]^+^, [M + Na]^+^, and [M – H]^−^ adducts, respectively, showed CCS deviations less than 2%. There are several reasons which may lead to this threshold being exceeded.

#### Protomers

The tendency for some compounds to form protomers can lead to different CCS values because different protonation sites can affect the shape and size of the molecules. As an example of this, ciprofloxacin has two competing protonation sites on its molecular structure, a carbonyl oxygen and an amine. Two different CCS values (173.3 and 185.3 Å^2^) were obtained for the [M + H]^+^ adduct of ciprofloxacin by Hines et al.,^16^ each reflecting a different site of protonation. The ^TW^CCS_N2_ value of the [M + H]^+^ adduct of ciprofloxacin determined here was 184.8 Å^2^. Only one CCS value was obtained here because ion mobility data were not sufficiently resolved, as shown on the mobility trace in Figure S5. Thus, a high CCS deviation of 6.2% (173.3 compared to 184.8 Å^2^) was obtained on comparing the ^TW^CCS_N2_ value for ciprofloxacin determined in this work against previously published CCS values for ciprofloxacin. The presence of multiple protomers also explains the high CCS deviation of theobromine. Two CCS values for the [M + H]^+^ adduct of theobromine, 131.1 and 138.9 Å^2^, were obtained in Nichols et al. (2018).^41^ The larger of these value had a deviation of 6.4% when compared to the value of 130.5 Å^2^ measured here.

#### Post-IMS Dissociation of a Noncovalent Cluster

Occasionally, a noncovalent cluster can form in the ion source, prior to entering the travelling wave device, which can subsequently undergo a dissociation after drifting through the device. When this happens, elevated CCS values are generated because the noncovalent clusters are larger than the target ions they contain. As an example of this, the [M + Na]^+^ adduct of chenodeoxycholic acid has a ^TW^CCS_N2_ value of 196.9 Å^2^. Two published CCS values for this compound are 202.8 Å^2^ from Poland et al.^39^ and 219.3 Å^2^ from Metabolic Profiling CCS Library.^38^ Multiple sites of protonation are not evident on the TWIMS platform. As such, the difference in the CCS values (196.9 compared to 219.3 Å^2^, ΔCCS% = 11.4%) may arise from the post-IMS dissociation of a noncovalent cluster.

It should be noted that, in some cases, the ion with highest abundance may not always yield the actual CCS value due to the presence of noncovalent clusters. The arrival time distribution (ATD) and mass spectrum of triclosan, a commonly used fungicide, are shown in Figure S6. The ion with the highest abundance had a measured ^TW^CCS_N2_ value of 177.5 Å^2^; however, three CCS values ranging from 157.3 to 160.0 Å^2^ were found for this compound in the literature.^28,29,40^ It can be seen from Figure S6 that a small peak with the arrival time of 4.52 ms has a ^TW^CCS_N2_ value of 157.4 Å^2^, which is in good agreement with published values. A careful examination of the mass spectra or comparing the experimental CCS values with those from the literature can avoid this kind of discrepancy.

#### IMS Calibration

^TW^CCS_N2_ values are obtained through appropriate calibration of the TWIMS platform and as such using calibration compounds with similar structural characteristics as the analytes to be investigated leads to increased accuracy of the measured ^TW^CCS_N2_ values.^49^ Therefore, performing the calibration using typical standards and over a limited data range may result in CCS deviations. For example, high CCS deviations were observed for the [M – H]^−^ adduct of *p*-coumaric acid (129.9 Å^2^) and ellagic acid (149.8 Å^2^). In the case of *p*-coumaric acid, four published CCS values were found, three of which range from 128.9 to 132.6 Å^2^,^38,41,50^ while the fourth, in the study by Gonzales et al.,^36^ has a value of 119.8 Å^2^, deviating in the region of −7.8% from our data.

Three published CCS values were found for ellagic acid: 152.0,^37^ 152.5,^40^ and 157.9 Å^2^,^36^ with the highest CCS deviation (5.4%) occurring, once again, between our data and those of Gonzales et al.^36^ Gonzales et al.^36^ calibrated their TWIMS system using deprotonated polyalanine standards. In our work, two fluoroalkanoic acids and some drug-like compounds were added to the polyalanines to extend the range over which the calibration was valid (see Table S2). These variations in the CCS measurements highlight the importance of establishing an appropriate CCS calibration strategy for the compounds to be analyzed on the TWIMS system. Recently, an improved CCS calibration approach has been proposed for the TWIMS system by Richardson and coworkers,^51^ which has the potential to further improve the accuracy the ^TW^CCS_N2_ measurements.

#### IMS Reproducibility

11% (33/300), 9.6% (11/114), and 26.9% (56/208) of the [M + H]^+^, [M + Na]^+^, and [M – H]^−^ adducts, respectively, had variations in their measured CCS values between 2 and 4%. This may be due to the reproducibility of the IMS measurements. A value of 2% is usually given for the variation in CCS measurements on TWIMS platforms. However, for some ions, the measurements may fall into the extreme ends of this ±2% tolerance, leading to elevated CCS discrepancies across different platforms and laboratories.

### Comparison to Predicted CCS Values from Machine Learning Approaches

**Figure 6:**
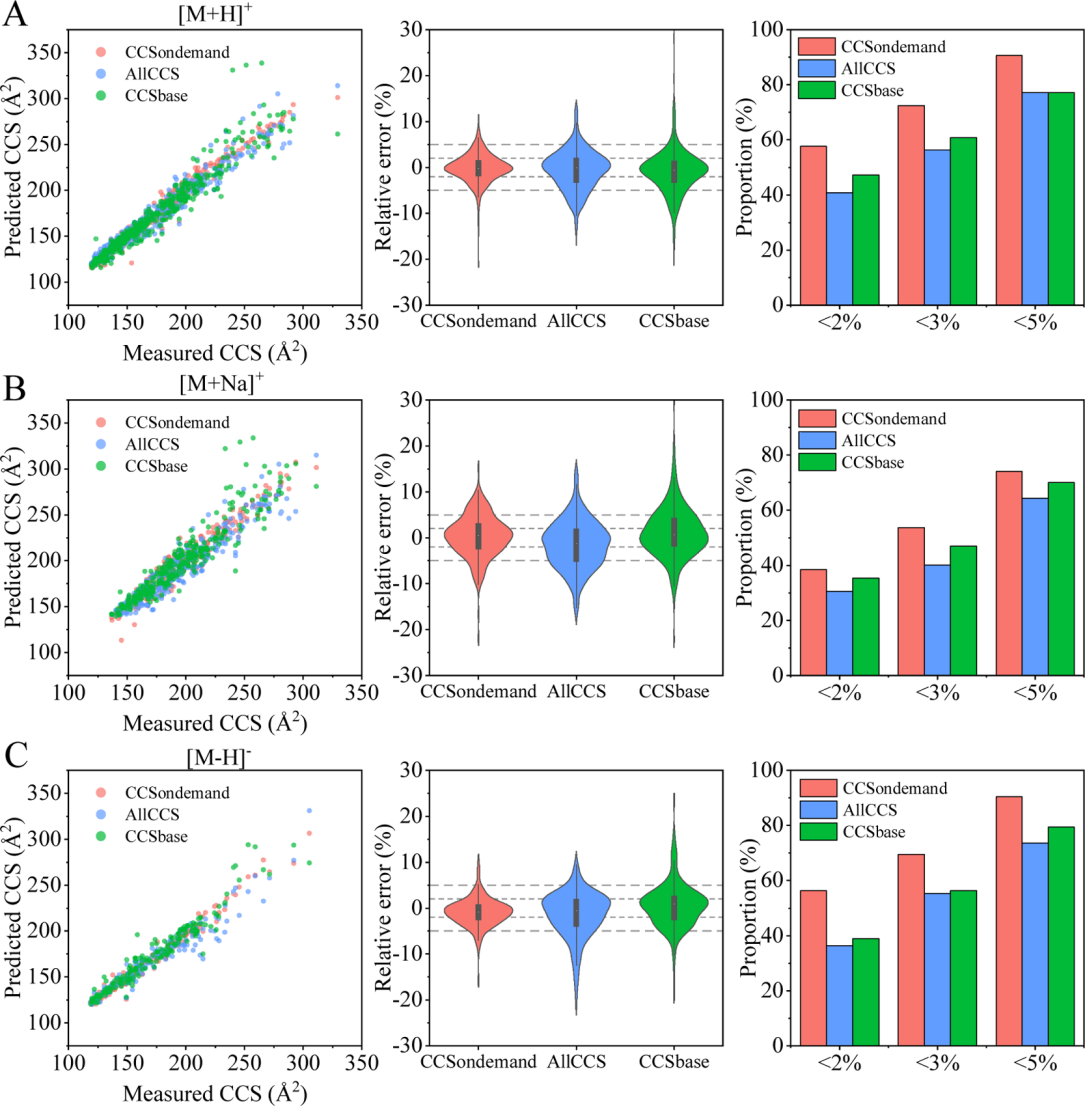
Comparison between experimental and predicted CCS values, (A) [M + H]^+^ adduct, (B) [M + Na]^+^ adduct, and (C) [M – H]^−^ adduct.

When no reference standard is available, comparing experimental CCS values to theoretical predictions can increase the confidence of identifications and reduce false positives.^32^ Although the experimental ^TW^CCS_N2_ values of 95 [M + H]^+^ and 64 [M + Na]^+^ have been compared with the predicted values from machine learning tools in a previous study,^35^ a more comprehensive comparison between experimental and predicted CCS values is still necessary. CCS values of 446 [M + H]^+^, 317 [M + Na]^+^, and 190 [M – H]^−^ adducts were predicted using three publicly available CCS prediction tools (CCSondemand, AllCCS and CCSbase) and compared to our experimental values. The proportions of experimental ^TW^CCS_N2_ records with relative deviations values less than 2, 3, and 5% from predicted values were compared. Evaluating the predictive performance of the prediction tools enabled an assessment of their applicability to FCCs. The results of the comparison are presented in Figure 6.

CCSondemand was found to be the most accurate CCS prediction tool for FCCs, followed by CCSbase and AllCCS. For CCSondemand, 57.6% of the predictions for [M + H]^+^ adducts and 56.3% of the predictions for [M – H]^−^ adducts agreed with the measured ^TW^CCS_N2_ values to within ±2%. On opening up the tolerance to 5, 90.6, and 90.5% of the predicted CCS values for [M + H]^+^ and [M – H]^−^ adducts, respectively, agreed with the measured data. The training data set for CCSondemand consisted of ^TW^CCS_N2_ values measured on Vion or Synapt platforms calibrated with the Major Mix IMS/ToF Calibration Kit as calibration mix.^31^ The same calibration kit was used to calibrate our instruments and this may explain why CCSondemand outperformed the other two tools in the prediction of CCS values of FCCs. Another reason for the more accurate prediction results provided by CCSondemand is that the training set of CCSondemand contains some experimental ^TW^CCS_N2_ values of FCCs, as mentioned previously.^35^

AllCCS and CCSbase predicted the CCS values of 40.8 and 47.3% of [M + H]^+^ adducts to within 2% of the measured values, respectively. The CCS values predicted by AllCCS and CCSbase for some compounds, commonly detected in FCMs, had relatively high errors when compared to the measured data. These included the primary aromatic amines (PAAs), as mentioned in the previous study,^35^ ultraviolet absorbers chimassorb 81 (−6.6 and −5.6%, respectively) and UV-360 (−12.6 and −8.2%, respectively). Oligomers, such as PPG5-PPG11, showed variations between the AllCCS and CCSbase predictions and measured values in the range of 5.4–12.7%. CCSbase predictions of the CCS values of cyclic PLA9 and cyclic PLA10 disagreed with the measured values by more than 30%. AllCCS predictions of the CCS values of antioxidants and their degradation products also showed elevated discrepancies to the measured data with Irgafos 168 (−11.6%), Irganox 1076 (−7.9%), 3,5-di-*tert*-butyl-4-hydroxybenzaldehyde (−9.1%), and 2,6-ditertbutyl-1,4-benzoquinone (−6.1%). Besides, it can be seen from Figure 6 that AllCCS and CCSbase present a trend toward negative prediction errors.

A possible explanation of the high deviations in the CCS values of AllCCS and CCSbase is that the training data sets did not contain many FCCs or FCC-like compounds.^35^ Additionally, the training data sets for AllCCS and CCSbase contained CCS values originating from both drift tube and travelling wave devices, and discrepancies have been shown to occur in CCS values measured on different instrument types. Hinnenkamp et al.^23^ has compared the ^TW^CCS_N2_ and drift tube CCS in nitrogen (^DT^CCS_N2_), finding that 7% protonated adducts and 13% sodium adducts have CCS deviations higher than 2%, this result indicates that CCS database cannot be used without caring their types. As the DTIMS can determine the CCS directly, without the need of calibration,^43^ an improved CCS calibration approach of TWIMS may be able to increase the consistency between experimental ^TW^CCS_N2_ and ^DT^CCS_N2_ values, thus leading to a more accurate CCS prediction model.

Predicted CCS values for [M + Na]^+^ adducts, for all three prediction tools, were relatively poor when compared to the measured values. CCSondemand once again provided the best results, but only 38.5% of the predicted CCS values for [M + Na]^+^ agreed with the measure data to within 2%. This may due to there being less measurements for [M + Na]^+^ adducts in the training data sets. Additionally, it is difficult to predict the CCS values of sodiated molecules using the molecular descriptors from neutral molecules.^35^

Considering the current accuracy of predicting CCS values, we believe that, at best, predicted CCS values can be used to help to eliminate false positives and to support tentative identifications. However, CCS prediction tools cannot be used to confirm the identification of an unknown compound. Connolly and coworkers have shown that predicted CCS values cannot accurately describe the difference of CCS values for isomeric glucuronide pairs.^52^ Technological developments are on-going though, and as the accuracy and reproducibility of experimentally obtained CCS values improves, a similar improvement can be expected in the accuracy of predicted CCS values.

In conclusion, a database of ^TW^CCS_N2_ values for extractable and leachable compounds from FCMs has been presented. This ^TW^CCS_N2_ database contains both IAS and NIAS. Excellent interday precision of the measured values has been shown, with all RSD values lower than 0.7%, indicating good reproducibility and stability of measurements from the TWIMS system. The ^TW^CCS_N2_ values in the database can serve as additional confirmation points for the identification of FCCs in targeted and untargeted screening analyses. It has also been argued that CCSondemand is a promising tool for the prediction of CCS values of FCCs, and the prediction performance of CCSondemand will be further improved by incorporating more high-quality CCS measurements in the training data set.

## Supporting Information Available

UPLC-IMS-QToF conditions; CCS calibration equations; calibration substances in the positive and negative ion modes; QC compounds; compounds for the interday precision of CCS measurement; experimental CCS values for QC compounds; regression equations of additives; CCS records with deviations higher than 5%; CCS deviations of QC compounds; structures of three photoinitiators; CCS versus *m*/*z* values for flame retardants, lubricants, and slip agents; interday precision; ATD of ciprofloxacin; and several CCS values of triclosan (PDF)
1038 CCS values from 675 standards (XLSX)

## Abbreviations

^TW^CCS_N2_: traveling wave collision cross section in nitrogen
FCMs: food contact materials
NIAS: nonintentionally added substances
FCCs: food contact chemicals
IAS: intentionally added substances
UPLC-QToF: ultrahigh performance liquid chromatography coupled to a quadrupole-time-of-flight mass spectrometer
GC-MS: gas chromatography-mass spectrometry
LC-MS: liquid chromatography-mass spectrometry
HRMS: high-resolution mass spectrometry
IMS: ion mobility spectrometry
DT: drift time
DIA: data-independent acquisition
RT: retention time
CCS: collision cross section
TWIMS: traveling wave ion mobility spectrometry
DTIMS: drift tube ion mobility spectrometry
PA: polyamide
PLA: polylactic acid
QC: quality control
RSDs: relative standard deviations
PFAS: perfluoroalkyl substances
BBP: benzyl butyl phthalate
DPP: diphenyl phthalate
FWHM: full width at half-maximum
*R*^2^: determination coefficient
BHT: butylated hydroxytoluene
PEG: polyethylene glycol
PPG: polypropylene glycol
ATD: arrival time distribution
PAAs: primary aromatic amines
^DT^CCS_N2_: drift tube collision cross section in nitrogen
