# Supplementary material for: A Collision Cross Section Database for Extractables and Leachables from Food Contact Materials
Source: J Agric Food Chem. 2022 Apr 5;70(14):4457–66. doi: 10.1021/acs.jafc.2c00724 (PMC9011387; doi:10.1021/acs.jafc.2c00724)
Supplement: Supplementary file 1 — jf2c00724_si_001.pdf [file jf2c00724_si_001.pdf]

Supporting Information

A Collision Cross Section (CCS) Database for Extractables and Leachables from Food Contact Materials

Xue-Chao Song[1], Elena Canellas[1], Nicola Dreolin[2], Jeff Goshawk[2], Cristina Nerin[1,*]

1. Department of Analytical Chemistry, Aragon Institute of Engineering Research I3A, CPS-University of Zaragoza, Maria de Luna 3, 50018, Zaragoza, Spain.

2. Waters Corporation, Altrincham Road, SK9 4AX, Wilmslow, United Kingdom.

* Corresponding author: Cristina Nerin, Phone: +34 976761873, Email: cnerin@unizar.es,.

Table of Contents:

Experimental Section: UPLC-IMS-QTof conditions.

Experimental Section: CCS Calibration of Vion IMS-QToF.

Table S1. Calibration substances in Major Mix IMS/Tof Calibration Kit and their CCS values in positive mode.

Table S2. Calibration substances in Major Mix IMS/Tof Calibration Kit and their CCS values in negative mode.

Table S3. Molecular formula, monoisotopic mass, retention time (RT) and $^{DT}CCS_{N2}$ of nine compounds in quality control (QC) solution.

Table S4. Thirty-eight compounds for validating the inter-day precision of CCS measurements.

Table S5. Comparison between reference and experimental CCS values for QC compounds for all batches of standards.

Table S6. The regression equations of different types of plasticizers, antioxidant, photoinitiators and oligomers.

Table S7. The CCS records with deviations higher than 5% with literature values.

Figure S1. CCS deviations of quality control (QC) compounds for all batches of standards, (A) positive ion mode and (B) negative ion mode.

Figure S2. Typical structures of benzophenone, thioxanthone and anthraquinone。

Figure S3. CCS values vs *m/z* values for flame retardants, lubricants and slip agents.

Figure S4. Inter-day precision of CCS measurement over two months.

Figure S5. Arrival time distribution (ATD) of ciprofloxacin.

Figure S6. Several CCS values of triclosan ($[M-H]^-$, *m/z* 286.9438) arising from post-IMS dissociation of noncovalent clusters.

Experimental Section

UPLC-IMS-QTof conditions. The chromatographic separation was performed using a CORTECS C18 column (2.1 × 100 mm, 1.6 μm particle size, 90 Å pore size) at a flow rate of 0.3 mL min^{-1}. Mobile phases were water (A) and methanol (B), both acidified with 0.1% of formic acid (v/v). The initial proportion of B was 5%, increased to 100% over 7 minutes, kept at 100% from 7 to 11 minutes, decreased to 5% over 0.1 minutes and re-conditioned until 13 minutes.

Data were acquired on the mass spectrometer in both positive and negative ion modes over the mass range of 50-1200 *m/z* with a scan time of 0.2 s. Electrospray ionization (ESI) conditions were as follows: capillary voltage, 1 kV; cone voltage, 30 V; source temperature, 120 ºC; desolvation temperature, 500 ºC; cone gas flow, 50 L h^{-1}; desolvation gas flow, 800 L h^{-1}. Data were acquired in high definition MS^E mode, with the instrument was switching between two collision energy states (low energy: 6 eV, high energy ramp: 20-40 eV) in order to obtain precursor and fragment ions within a single acquisition. Leucine-Enkephalin ($[M+H]^+$, *m/z* 556.2766 and $[M-H]^-$, *m/z* 554.2620) at a concentration of 100 ng/mL was infused at a rate of 15 μL/min for real-time mass correction. IM separations were performed with a travelling wave velocity of 250 m/s and IMS pulse height of 45 V, N_2 was used as the drift gas at a flow of 25 mL/min. The Vion platform works at a room temperature of 25 ºC.

CCS Calibration of Vion IMS-QToF. In Vion platform, the CCS calibration was preformed automatically in UNIFI software. The calibration process was described by the following functions, firstly, the normalized CCS Ω_n was calculated from the reference CCS values Ω of the calibrates (the reference CCS values of calibrates are detected by drift tube ion mobility spectrometry (DTIMS) using a step-field method):

$$\Omega_n = \Omega \times \frac{\sqrt{\mu}}{z}$$

where $\mu = \frac{m_{ion} \times m_{gas}}{m_{ion} + m_{gas}}$, m_{ion} and m_{gas} are the mass of each calibrate and drift gas (N_2), respectively. Then the arrival time (or drift time) t_A of the calibrates were recorded and the corrected arrival time t_d was calculated:

$$t_d = t_A - c\sqrt{\frac{m}{z}}$$

where $c = \frac{EDC}{1000}$, *EDC* is the enhanced duty cycle (EDC) delay coefficient of the instrument. After this, the normalized CCS Ω_n versus t_d are fitted using an empirically-derived power-law function:

$$\Omega_n = A(t_d - t_0)^B$$

t_0 is the undetermined time off-set, the coefficient *A* and exponential factor *B* are calculated, which will be used for the derivation of CCS of unknown compounds. This function was then used to calculate the CCS values of calibrates and compared with their reference CCS, the acceptance criteria of the calibration is that CCS deviation of each calibrate is within ±2%.

More information about CCS calibration approach can be seen in Righetti et al. (2020)[1] and Bush et al. (2010).[2]

Table S1. Calibration substances in Major Mix IMS/Tof Calibration Kit and their CCS values in positive mode.

Compound	Formula	m/z $[M+H]^+$	CCS ($Å^2$)
Acetaminophen	C8H9NO2	152.0706	130.4
Reserpine fragment	C8H10N4O2	195.0877	138.2
Sulfaguanidine	C7H10N4O2S	215.0597	146.8
Sulfadimethoxine	C12H14N4O4S	311.0809	168.4
Val-Tyr-Val	C19H29N3O5	380.2180	191.7
Verapamil	C27H38N2O4	455.2904	208.8
Terfenadine	C32H41NO2	472.3210	228.7
Polyalanine, n=7	C21H37N7O8	516.2776	211.0
Leucine Enkephalin	C28H37N5O7	556.2766	229.8
Polyalanine, n=8	C24H42N8O9	587.3148	228.0
Reserpine	C33H40N2O9	609.2807	252.3
Polyalanine, n=9	C27H47N9O10	658.3519	243.0
Polyalanine, n=10	C30H52N10O11	729.3890	256.0
Polyalanine, n=11	C33H57N11O12	800.4261	271.0
Polyalanine, n=12	C36H62N12O13	871.4632	282.0
Polyalanine, n=13	C39H67N13O14	942.5003	294.0
Polyalanine, n=14	C42H72N14O15	1013.5374	306.0
Polyalanine, n=15	C45H77N15O16	1084.5746	321.5
Polyalanine, n=16	C48H82N16O17	1155.6117	333.6
Ultramark 1621	C20H18O6N3P3F28	1022.0034	263.1
Ultramark 1621	C22H18O6N3P3F32	1121.9970	276.5

Table S2. Calibration substances in Major Mix IMS/Tof Calibration Kit and their CCS values in negative mode.

Compound	Formula	m/z $[M-H]^-$	CCS (Å^2)
Acetaminophen	C8H9NO2	150.0561	131.5
Theophylline	C7H7N4O2	179.0575	132.4
Sulfaguanidine	C7H10N4O2S	213.0452	145.2
Sulfadimethoxine	C12H14N4O4S	309.0663	170.1
Val-Tyr-Val	C19H29N3O5	378.2034	192.5
Leucine Enkephalin	C28H37N5O7	554.2620	225.3
Perfluoroheptanoic acid -CO2	C6F13	318.9798	130.1
Perfluorooctanoic acid -CO2	C7F15	368.9766	137.2
Polyalanine, n=8	C24H42N8O9	585.3002	227.7
Reserpine	C33H40N2O9	607.2661	265.2
Polyalanine, n=9	C27H47N9O10	656.3373	242.1
Polyalanine, n=10	C30H52N10O11	727.3744	255.9
Polyalanine, n=11	C33H57N11O12	798.4115	268.5
Polyalanine, n=12	C36H62N12O13	869.4487	280.2
Polyalanine, n=13	C39H67N13O14	940.4858	294.6
Polyalanine, n=14	C42H72N14O15	1011.5228	308.8
Polyalanine, n=15	C45H77N15O16	1082.5600	322.4
Ultramark 1621	C23H20O8N3P3F32	1165.9880	275.8

Table S3. Molecular formula, monoisotopic mass, retention time (RT) and $^{DT}CCS_{N2}$ of nine compounds in quality control (QC) solution.

Compound	Molecular formula	Monoisotopic mass (Da)	RT (min)	CCS (Å^2) $[M+H]^+$	CCS (Å^2) $[M-H]^-$
Acetaminophen	$C_8H_9NO_2$	151.0633	1.96	130.4	131.5
Caffeine	$C_8H_{10}N_4O_2$	194.0804	2.76	138.2	-
Sulfaguanidine	$C_7H_{10}N_4O_2S$	214.0524	1.02	146.8	145.2
Sulfadimethoxine	$C_{12}H_{14}N_4O_4S$	310.0736	3.83	168.4	170.1
Val-tyr-val	$C_{19}H_{29}N_3O_5$	379.2107	2.87	191.7	192.5
Verapamil	$C_{27}H_{38}N_2O_4$	454.2832	4.57	208.8	-
Terfenadine	$C_{32}H_{41}NO_2$	471.3137	5.64	228.7	-
Leucine-enkephalin	$C_{28}H_{37}N_5O_7$	555.2693	3.97	229.8	225.3
Reserpine	$C_{33}H_{40}N_2O_9$	608.2734	4.92	252.3	265.2

Table S4. Thirty-eight compounds for validating the inter-day precision of CCS measurement.

No.	Compounds	PubChem CID	ESI (+)	ESI (-)
1	Stearic acid	5281		•
2	Antioxidant 425	6928	•	•
3	N-(4-Hydroxyphenyl)Stearamide	7689	•	•
4	Antioxidant 2246	8398	•	•
5	4-Hydroxybenzophenone	14347	•	•
6	11-Aminoundecanoic acid	17083	•	•
7	Irganox 1024	61916	•	•
8	Irganox 1035	64883	•	•
9	12-Aminododecanoic acid	69661	•	•
10	MOPS	70807	•	•
11	Tioxolone	72139	•	•
12	3,5-Di-tert-butyl-4-hydroxybenzaldehyde	73219	•	•
13	3-(3,5-Di-tert-butyl-4-hydroxyphenyl)propionic acid	88389	•	•
14	Antioxidant 1790	93221	•	•
15	UV-234	112412	•	•
16	Ethyl 2-cyano-3,3-diphenylacrylate	243274	•	
17	3,5-Di-tert-butyl-4-hydroxyacetophenone	616296	•	•
18	Bisphenol A bis(3-chloro-2-hydroxypropyl) ether	3479589	•	•
19	Bisphenol F bis(2,3-dihydroxypropyl) ether	3928015	•	•
20	Bisphenol A (3-chloro-2-hydroxypropyl) (2,3-dihydroxypropyl) Ether	4166922	•	•
21	2,2'-Dihydroxy-4-methoxybenzophenone	8569	•	•
22	Erucamide	5365371	•	
23	Dibutyl phosphate	7881	•	•
24	Methylparaben	7456		•
25	Propylparaben	7175		•
26	Diethyl phthalate	6781	•	
27	Tinuvin P	17113	•	
28	Dibutyl sebacate	7986	•	
29	Diphenyl phthalate	6778	•	
30	Tinuvin 327	77470	•	•
31	Tri(p-cresyl) phosphate	6529	•	
32	Uvitex OB	292429	•	
33	Cyasorb 2908	94623	•	•
34	Irganox 1076	16386	•	•
35	Irganox 245	91620	•	•
36	Irganox 1098	90004	•	•
37	Tinuvin 360	3571576	•	•
38	Irganox 1330	74370	•	•

Table S5. Comparison between reference and experimental CCS values for QC compounds for all batches of standards.

	Compounds	Adducts	m/z	CCS_{ref} (Å^2)	CCS_{exp} ± SD (Å^2)	RSD (%)	error (%)
Positive	Acetaminophen	$[M+H]^+$	152.0706	130.4	131.8 ± 1.1	0.8	1.1
(n = 76)	Caffeine	$[M+H]^+$	195.0877	138.2	137.7 ± 1.0	0.7	-0.4
	Sulfaguanidine	$[M+H]^+$	215.0597	146.8	146.5 ± 0.9	0.6	-0.2
	Sulfadimethoxine	$[M+H]^+$	311.0809	168.4	168.0 ± 1.0	0.6	-0.3
	Val-tyr-val	$[M+H]^+$	380.2180	191.7	193.6 ± 1.0	0.5	1.0
	Verapamil	$[M+H]^+$	455.2904	208.8	210.2 ± 1.2	0.6	0.7
	Terfenadine	$[M+H]^+$	472.3210	228.7	231.0 ± 1.5	0.6	1.0
	Leucine-enkephalin	$[M+H]^+$	556.2766	229.8	228.9 ± 1.3	0.6	-0.4
	Reserpine	$[M+H]^+$	609.2807	252.3	252.3 ± 1.6	0.6	-0.0
Negative	Acetaminophen	$[M-H]^-$	150.0561	131.5	130.2 ± 0.9	0.7	-1.0
(n = 24)	Sulfaguanidine	$[M-H]^-$	213.0452	145.2	144.1 ± 0.9	0.7	-0.8
	Sulfadimethoxine	$[M-H]^-$	309.0663	170.1	170.4 ± 1.1	0.6	0.2
	Val-tyr-val	$[M-H]^-$	378.2034	192.5	194.1 ± 1.2	0.6	0.9
	Leucine-enkephalin	$[M-H]^-$	554.2620	225.3	223.5 ± 1.6	0.7	-0.8
	Reserpine	$[M-H]^-$	607.2661	265.2	267.9 ± 1.5	0.5	1.0

Table S6. The regression equations of different types of plasticizers, antioxidant, photoinitiators and oligomers.

Additives	Types	Number of CCS	Equations	R^2
Plasticizers	Phthalates	30	$y = 5.6996x^{0.6041}$	0.9610
	Adipates and sebacates	10	$y = 6.0367x^{0.5975}$	0.9652
	Citrates	5	$y = 6.5292x^{0.5732}$	0.8924
	Others	12	$y = 6.6809x^{0.5745}$	0.9837
Antioxidants	Phenolics	34	$y = 13.2458x^{0.4601}$	0.9363
	Phosphites	6	$y = 8.5852x^{0.5358}$	0.9299
	Degradation products	23	$y = 18.5045x^{0.3994}$	0.6752
	Others	4	$y = 6.4174x^{0.5878}$	0.9820
Photoinitiators	Benzophenones	15	$y = 4.8925x^{0.6317}$	0.9191
	Amine co-initiators	4	$y = 5.7303x^{0.6154}$	0.9780
	Phosphine oxides	4	$y = 50.3439x^{0.2289}$	0.0754
	Thioxanthones	4	$y = 4.0913x^{0.6512}$	0.7309
	Anthraquinones	6	$y = 23.7164x^{0.3320}$	0.9372

Oligomers	Adhesive oligomers	7	$y = 10.1334x^{0.4889}$	0.9785
	PA	16	$y = 96.013 + 0.244x$	0.9933
	PLA	42	$y = 7.5203x^{0.5306}$	0.9749
	PEG and PPG	65	$y = 92.801 + 0.2296x$	0.9843

y represents CCS values; x represents *m/z* of ions.

Table S7. The CCS records with deviations higher than 5% with literature values.

No.	Name	Adducts	PubChem CID	CCS in our database (Å^2)	CCS in literature (Å^2)	Deviations (%)
1	Ferulic acid	$[M+H]^+$	445858	141.07	128.8[3]	-8.7
2	Ciprofloxacin	$[M+H]^+$	2764	184.76	173.3[3]	-6.2
3	Glycocholic acid	$[M+H]^+$	10140	190.82	200.4[4]	5.02
4	Dexamethasone	$[M+H]^+$	5743	183.45	192.8[5]	5.10
5	Theobromine	$[M+H]^+$	5429	130.54	138.3[6]	5.94
6	Glycocholic acid	$[M+H]^+$	10140	190.82	205.2[3]	7.54
7	Chenodeoxycholic acid	$[M+Na]^+$	10133	196.88	219.3[5]	11.39
8	p-Coumaric acid	$[M-H]^-$	637542	129.92	119.8[7]	-7.79
9	Dehydrocholic acid	$[M-H]^-$	6674	208.37	192.4[8]	-7.66
10	Arbutin	$[M-H]^-$	440936	172.99	160.81[6]	-7.04
11	(+)-Lactose	$[M-H]^-$	6134	176.62	166.7[9]	-5.62
12	(+)-Lactose	$[M-H]^-$	6134	176.62	167.0[5]	-5.45
13	D-(+)-Trehalose	$[M-H]^-$	7427	175.09	166.0[5]	-5.19
14	Lithocholic acid	$[M-H]^-$	9903	209.67	198.9[8]	-5.14
15	Stearic acid	$[M-H]^-$	5281	183.84	174.42[9]	-5.12
16	Hyodeoxycholic acid	$[M-H]^-$	5283820	209.77	199.2[8]	-5.04
17	Ellagic acid	$[M-H]^-$	5281855	149.78	157.9[7]	5.42

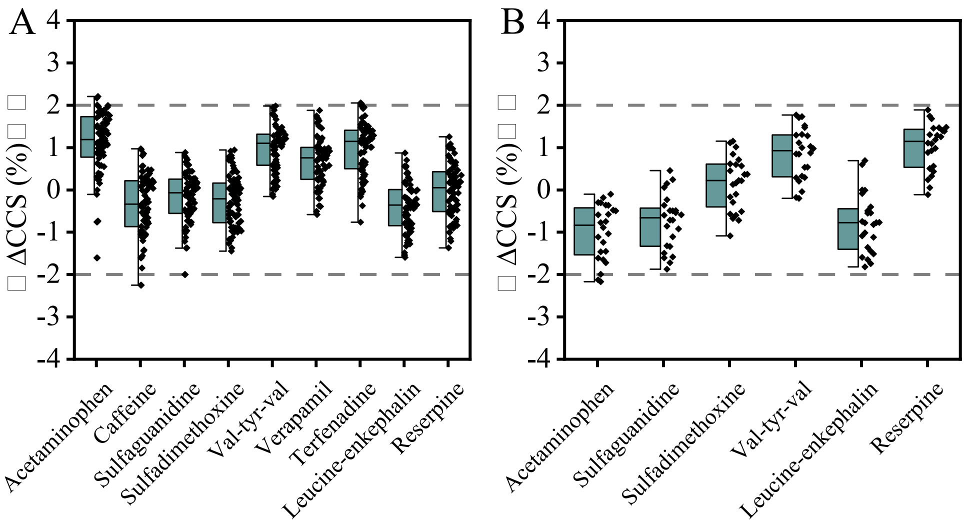

Figure S1. CCS deviations of quality control (QC) compounds for all batches of standards, (A) positive ion mode and (B) negative ion mode.

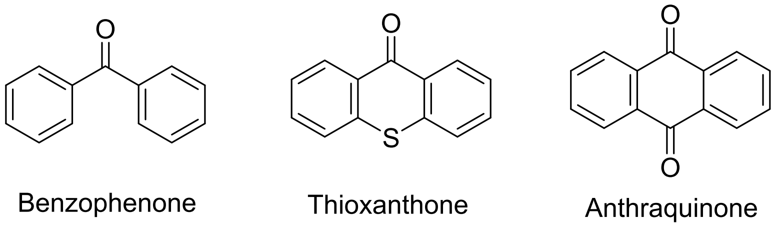

Figure S2. Typical structures of benzophenone, thioxanthone and anthraquinone.

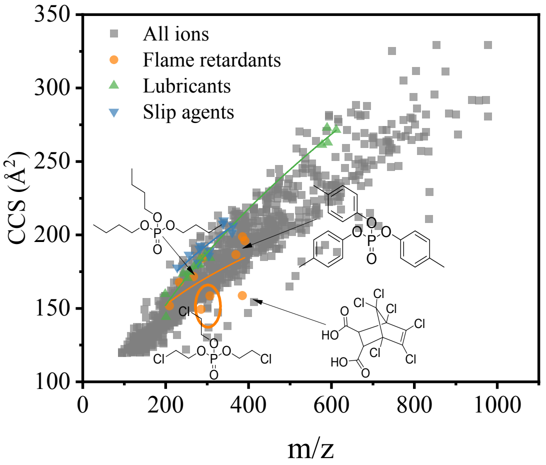

Figure S3. CCS values vs *m/z* values for flame retardants, lubricants and slip agents.

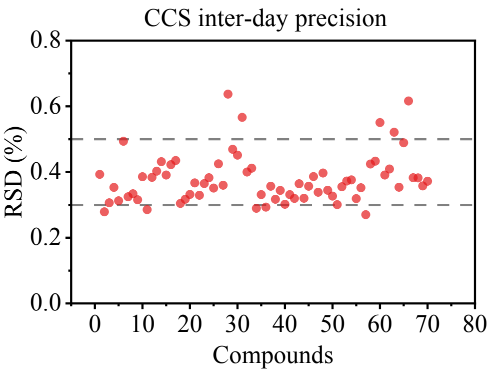

Figure S4. Inter-day precision of CCS measurement over two months.

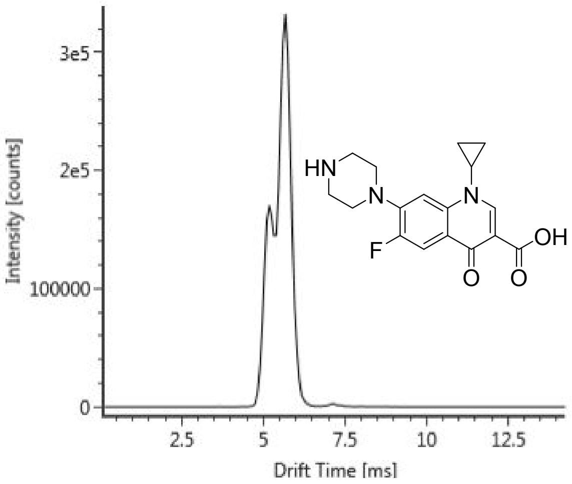

Figure S5. Arrival time distribution (ATD) of ciprofloxacin.

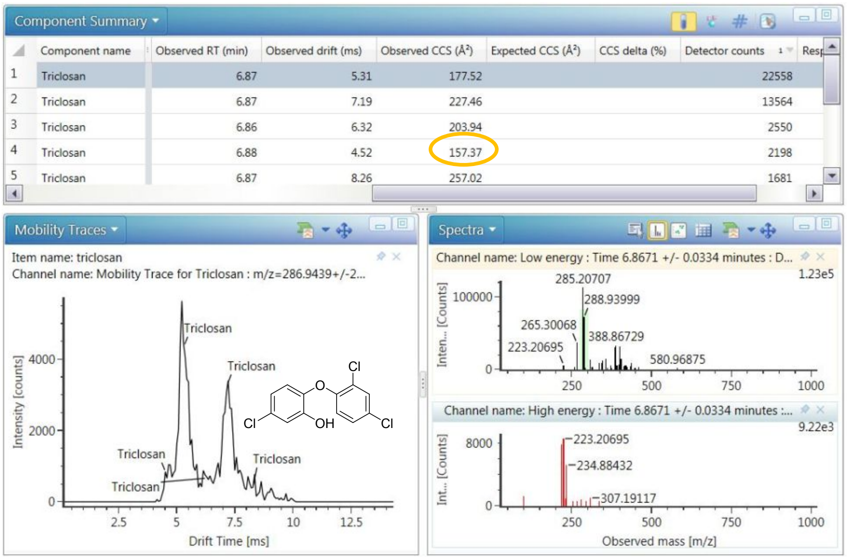

Figure S6. Several CCS values of triclosan ([M-H]$^{-}$, m/z 286.9438) arising from post-IMS

dissociation of noncovalent clusters.

References

1. Righetti, L.; Dreolin, N.; Celma, A.; McCullagh, M.; Barknowitz, G.; Sancho, J. V.; Dall'Asta, C., Travelling Wave Ion Mobility-Derived Collision Cross Section for Mycotoxins: Investigating Interlaboratory and Interplatform Reproducibility. *J. Agric. Food Chem.* 2020, 68, (39), 10937-10943.
2. Bush, M. F.; Hall, Z.; Giles, K.; Hoyes, J.; Robinson, C. V.; Ruotolo, B. T., Collision cross sections of proteins and their complexes: a calibration framework and database for gas-phase structural biology. *Anal. Chem.* 2010, 82, (22), 9557-9565.
3. Hines, K. M.; Ross, D. H.; Davidson, K. L.; Bush, M. F.; Xu, L., Large-Scale Structural Characterization of Drug and Drug-Like Compounds by High-Throughput Ion Mobility-Mass Spectrometry. *Anal. Chem.* 2017, 89, (17), 9023-9030.
4. Nichols, C. M.; Dodds, J. N.; Rose, B. S.; Picache, J. A.; Morris, C. B.; Codreanu, S. G.; May, J. C.; Sherrod, S. D.; McLean, J. A., Untargeted Molecular Discovery in Primary Metabolism: Collision Cross Section as a Molecular Descriptor in Ion Mobility-Mass Spectrometry. *Anal. Chem.* 2018, 90, (24), 14484-14492.
5. Waters Corporation, Metabolic Profiling CCS Library. URL https://marketplace.waters.com/apps/177290/metabolic-profiling-ccs-library#!overview. (accessed 10 March 2022).
6. Waters Corporation, Natural Products Profiling CCS Library. URL https://marketplace.waters.com/apps/255816/naturalproducts-profiling-ccs-library#!overview. (accessed 10 March 2022).
7. Gonzales, G. B.; Smagghe, G.; Coelus, S.; Adriaenssens, D.; De Winter, K.; Desmet, T.; Raes, K.; Van Camp, J., Collision cross section prediction of deprotonated phenolics in a travelling-wave ion mobility spectrometer using molecular descriptors and chemometrics. *Anal. Chim. Acta* 2016, 924, 68-76.
8. Poland, J. C.; Leaptrot, K. L.; Sherrod, S. D.; Flynn, C. R.; McLean, J. A., Collision Cross Section Conformational Analyses of Bile Acids via Ion Mobility-Mass Spectrometry. *J. Am. Soc. Mass Spectrom.* 2020, 31, (8), 1625-1631.
9. Zhou, Z.; Shen, X.; Tu, J.; Zhu, Z. J., Large-Scale Prediction of Collision Cross-Section Values for Metabolites in Ion Mobility-Mass Spectrometry. *Anal. Chem.* 2016, 88, (22), 11084-11091.
